# Supplementary material for: Transmission of natural scene images through a multimode fibre
Source: Nat Commun. 2019 May 2;10:2029. doi: 10.1038/s41467-019-10057-8 (PMC6497636; doi:10.1038/s41467-019-10057-8)
Supplement: Supplementary file 1 — Supplementary Information [file 41467_2019_10057_MOESM1_ESM.pdf]

Transmission of natural scene images through a multimode fibre: Supplementary Material.

Piergiorgio Caramazza[1], Oisín Moran[2], Roderick Murray-Smith[2], Daniele Faccio[1]
[1]School of Physics and Astronomy, University of Glasgow, Glasgow, G12 8QQ, UK
[2]School of Computing Science, University of Glasgow, Glasgow, G12 8QQ, UK*

Supplementary information to "Transmission of natural scene images through a multimode fibre" providing additional details regarding the code and measurements.

SUPPLEMENTARY NOTE 1: EFFECT OF IMAGE SIZE AT FIBRE INPUT.

Whilst performing experiments we investigated the effect of different focusing (i.e. image size) configurations at the fibre input. We performed three different experiments in which we used exactly the same data/images for the retrieval of the W matrix and also the same test images. However, by inserting an additional telescope straight after the SLM, we could control the images sizes before the objective coupling into the fibre. By demagnifying the input images by factors 1x, 1.4x and 4x, we expect the angular spread with which the images are being focused to decrease. In these three cases, an effective numerical aperture can be estimated by considering the objective focal length ($f = 34$ mm) and the image size at the input of the fiber. Respectively, the sides of the images are: 10.5 mm, 7.5 mm and 2.6 mm, corresponding to $\text{NA}_{eff} = 0.22$, $\text{NA}_{eff} = 0.16$ and $\text{NA}_{eff} = 0.05$. In all three case, the images were placed in a similar position on the fibre, i.e. slightly off to the centre. Indeed, we noticed that placing the image directly in the centre of the fibre led to very clear accumulation of the speckles patterns towards the centre of the fibre, surrounded by broken ring-like structures in the outer region. These ring-like structures are a clear indication of higher modes, in keeping with previous reports also from other groups [1]. However, the fact that these modes maintain their ring-like

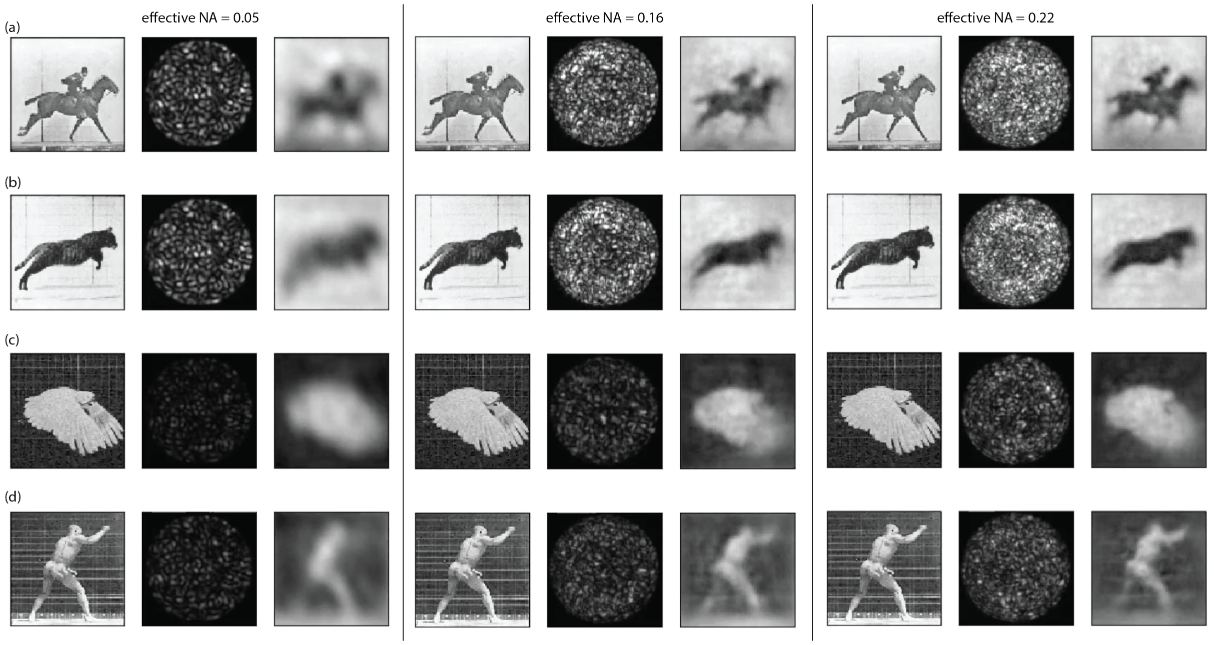

Supplementary Figure 1. Comparison between same images focused with different angular spread reported by an estimation of the effective NA (this was realized by putting a telescope before the objective with different magnification). Each image (a)-(d) is reported along with its relative speckle pattern at the fiber output and ANN reconstruction. An increase in the effective NA leads to averaged smaller speckles (indicating that higher spatial frequency modes have been excited).

* Daniele.Faccio@glasgow.ac.uk,
Roderick.Murray-Smith@glasgow.ac.uk

structure as opposed to a more random speckle structure indicates that only a few of the higher order modes have been excited. Conversely, the full speckle pattern, distributed across the full fibre output, is obtained only by exciting many modes. This condition was observed by displacing the beam slightly to either side with respect to the central position. This is the desired configuration as the objective here is to image with the highest resolution possible: the fine features of any image are carried by the higher spatial frequencies, which in turn correspond to the higher order modes in the fibre.
The effect of changing the inout focusing condition can be clearly seen in Fig. 1. When focusing with the smallest NA_{eff} at the fibre input facet, the speckles at the output are largest, corresponding to fewer modes and the final retrieval is significantly worse when compared to the largest NA_{eff}. The intermediate NA_{eff} shows slightly worse results with respect to the largest NA_{eff}. A consistent trend is therefore found between the effective NA, the size and number of speckles at the fibre output and final image quality.

SUPPLEMENTARY NOTE 2: METHODS FOR IMAGE COMPARISON

Two methods have been considered in order to quantify the quality of our predicted images: the structural similarity index (SSIM) [2] and the Parson correlation coefficient (PCC). In both cases, a perfect match would correspond to the maximum value 1. Considering two images X and Y, we use the definitions:

$$SSIM(X,Y) = \frac{(2\mu_X\mu_Y + C_1)(2\sigma_{XY} + C_2)}{(\mu_X^2\mu_Y^2 + C_1)(\sigma_X^2\sigma_Y^2 + C_2)} \tag{1}$$

where μ_X represent the average of X, μ_Y the average of Y, σ_{XY} the covariance of X and Y, σ_X^2 the variance of X and σ_Y^2 the variance of Y. Whereas, C_1 and C_2 are two parament defined as $C_1 = (K_1L) * *2$ and $C_1 = (K_2L) * *2$ where K_1 was set to 0.01 and K_2 to 0.03 and L is the dynamic range of the image pixels. Instead, the Parson correlation coefficent is defined as:

$$PCC(X,Y) = \frac{\sum_i(x_i - \bar{X})(y_i - \bar{Y})}{\sqrt{\sum_i(x_i - \bar{X})^2\sum_i(y_i - \bar{Y})^2}} \tag{2}$$

where x_i and y_i indicate the pixels with index i respectively of the images X and Y, and $\bar{X}$ (similarly with Y) the average of X.

SUPPLEMENTARY NOTE 3: DATA

For optimisation and testing of the model parameters, we use 50,000 images from the ImageNet collection [3]. The training and experimental datasets are supplied as additional material [4]. Validation image examples are images and videos from the Muybridge collection such as a running horse, a jumping cat and a flying parrot. As explained in the main text, the behaviour of the image retrieval seems to be largely independent of the actual test images that was chosen. We focused mainly on the Muybridge images in the main text but here show other examples in Fig. 2. Figures 2(a) and (b) show colour photographs of the University of Glasgow, imaged through a 1 m fibre. (c) is a grayscale image of a panda. (d) is a satellite image of the Earth imaged through a 10 m fibre at a two different times after the initial training an inversion process is completed showing that the retrieved inversion matrix and setup can still be used at a later time, as would be expected (yet still need to be verified) for a robust inversion system. In (e) we report the speckle patterns relative to (d) at different times (1 hour, 16 hours, 40 hours and 52 hours) of a single channel of the RGB image. In order to allow a quantitative comparison, we used the SSIM defined in the previous section. We note that the setup was not placed in a specifically engineered or stabilised environment and was thus subject to standard night-day temperature fluctuations (2-3 degrees) and environmental vibrations. On the other hand, as we can see in Fig. 2-(e), the correlation between the speckle pattern in time is still high, allowing a good reconstruction. We judged out the scope of the present work to introduce models able to deal with deep changes in the system transmission matrix, such as in presence of relevant bending or temperature variations. Indeed, in future work it would be interesting to explore the possibilities given by a physics inspired artificial neural network also respect to these challenges.
In Fig. 3 we show a collection of images taken from the ImageNet database together with their respective output speckle patterns (at the output of 1 m long fibre) and final reconstructions. These images provide further evidence for the image variability and robustness of the imaging reconstruction.

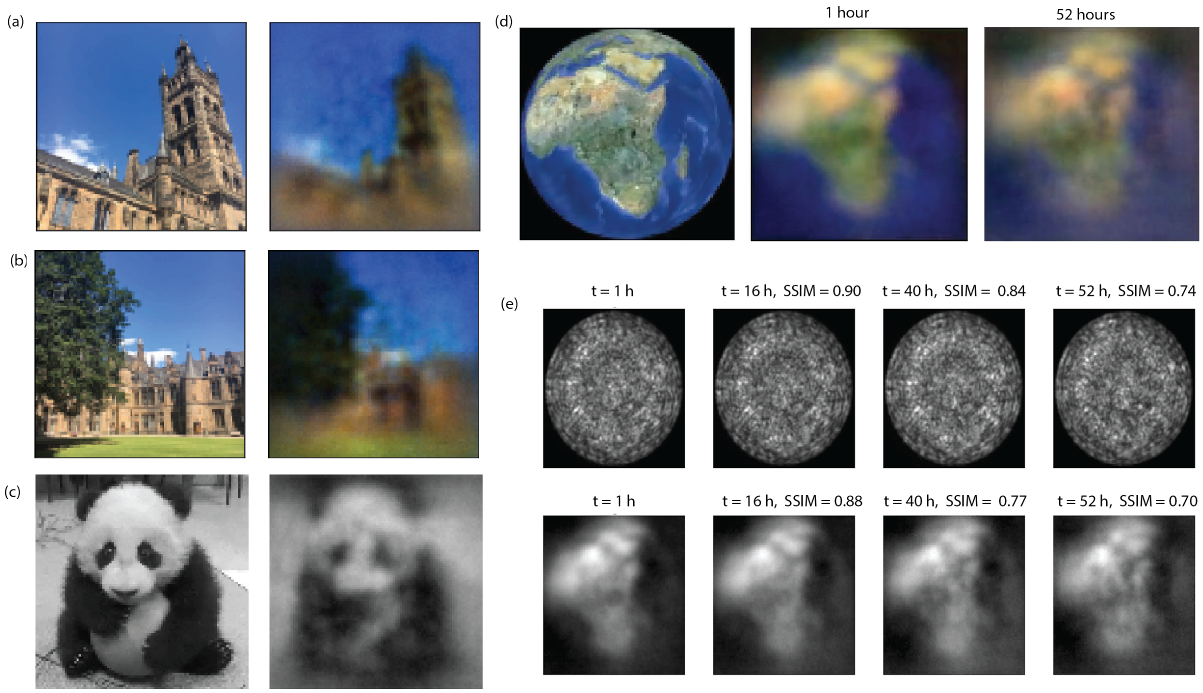

Supplementary Figure 2. Additional examples of natural scenes imaged through a mulitmode fiber. (a)-(b) Colour photographs of Glasgow University (imaged through a 1 meter fibre). (c) Black and white photograph of a panda (imaged through 10 m fibre). (d) Satellite photograph of Earth imaged at two different times after the transmission of the initial training dataset (1 hour and 52 hours, imaged through 10 m fibre). (e) Comparison between green-colour-channel speckles of the images in (d), at different times. The speckle patterns at time 1 hour, 16 hours, 40 hours and 52 hours are shown on the first column together with the SSIM parameters calculated respect to the time "1 hour" speckle pattern. As can be seen, the 10 m length of the fibre leads to a slow decorrelation of the speckle pattern, quantified here by the decreasing SSIM with time. Similarly, the reconstructed green-channel-images show a slow degradation of the SSIM with a linear dependence with respect to the speckle SSIM degradation.

SUPPLEMENTARY NOTE 4: SOFTWARE

The code was developed in Python 3.6.5 with a standard Anaconda `http://www.anaconda.com/download` configuration, including Keras [5] and TensorFlow [6]. The code is supplied as additional supplementary material that can be downloaded together with all of the training and experimental data/images [4].

A. Model specification

The model is implemented as a simple complex, densely connected layer. The individual weights are regularised with an L_2 minimising term, weighted by $\lambda = 0.03$. weights are initialised randomly, uniformly between ± 0.002. Recorded images are collected into a training set of $N = 45,000$ and validation set of $5,000$.

Listing 1. Model specification

```
speckle_dim = 120
out_dim = 92
lamb = 0.03

inp = Input(shape=(speckle_dim**2,2))
comp = ComplexDense(out_dim**2, use_bias=False,
                    kernel_initializer=RandomUniform(-.002, .002),
                    kernel_regularizer=regularizers.l2(lamb))(inp)
amp = Amplitude()(comp)
```

```
model = Model(inputs=inp, outputs=amp)
```

The ComplexDense Layer is a custom layer we developed for Keras. It is a straightforward Dense layer, but with complex-valued weights. The complex weights are represented with Complex64 64-bit data types. Its only task is to implement the complex-valued multiplication.

Listing 2. Custom ComplexDense Layer

```
class ComplexDense(Layer):

def __init__(self, output_dim,
             activation=None,
             use_bias=True,
                 kernel_initializer='glorot_uniform',
             bias_initializer='zeros',
             kernel_regularizer=None,
             **kwargs):
        super(ComplexDense, self).__init__(**kwargs)
    self.output_dim = output_dim
    self.activation = activations.get(activation)
    self.use_bias = use_bias
    self.kernel_initializer = initializers.get(kernel_initializer)
    self.bias_initializer = initializers.get(bias_initializer)
    self.kernel_regularizer = regularizers.get(kernel_regularizer)


def build(self, input_shape):
    self.kernel = self.add_weight(name='kernel',
                                  shape=(input_shape[1], self.output_dim, 2),
                                  initializer=self.kernel_initializer,
                                  regularizer=self.kernel_regularizer,
                                  trainable=True)

    if self.use_bias:
        self.bias = self.add_weight(name='bias',
                                   shape=(self.output_dim, 2),
                                   initializer=self.bias_initializer,
                                   trainable=True)
    else:
        self.bias = None
    super(ComplexDense, self).build(input_shape)

def call(self, X):
    # True Complex Multiplication (by channel combination)
    complex_X = channels_to_complex(X)
    complex_W = channels_to_complex(self.kernel)

    complex_res = complex_X @ complex_W

    if self.use_bias:
        complex_b = channels_to_complex(self.bias)
             = K.bias_add(complex_res, complex_b)

    output = complex_to_channels(complex_res)

    if self.activation is not None:
        output = self.activation(output)

    return output
```

```
def compute_output_shape(self, input_shape):
    return (input_shape[0], self.output_dim, 2)

def get_config(self):
    config = {'output_dim': self.output_dim,
              'use_bias': self.use_bias,
              'kernel_initializer':
                 initializers.serialize(self.kernel_initializer),
              'bias_initializer': initializers.serialize(self.bias_initializer),
              'kernel_regularizer':
                 regularizers.serialize(self.kernel_regularizer)
              }
    base_config = super(ComplexDense, self).get_config()
    return dict(list(base_config.items()) + list(config.items()))
```

B. Parameter optimisation

The model fitting uses the standard Keras routines, involving a choice of stochastic gradient descent and mean square error for the cost function. Here the data variable *x_train, y_train, x_validation, y_validation, x_text and y_test* refer to the amplitudes of, respectively, speckle patterns (x) and original images (y).

Listing 3. Compile and optimise parameters

```
model.compile(optimizer=SGD(lr=1e-5), loss='mse', metrics=['mse'])
model_chk = ModelCheckpoint(weights_filepath, monitor='mse', verbose=0,
                            save_best_only=False,
                            save_weights_only=False, mode='auto', period=1)
reduce_lr = ReduceLROnPlateau(monitor='loss', factor=0.1, patience=2,
            min_lr=lr/1e3, verbose=1,)
early_stop = EarlyStopping(monitor='loss', min_delta=0.0001, patience=8)

model.fit(x_train, y_train, validation_data = (x_validation, y_validation),
          epochs = 850, batch_size = 32,
          callbacks = [model_chk, reduce_lr, early_stop], shuffle = True)
```

Once the network parameters have converged (we ran the network for 850 iterations which takes ca 2 days on a PC with Nvidia TitanXp GPU card, you can generate predictions of outputs using

```
pred_test = model.predict(x_test)**2
```

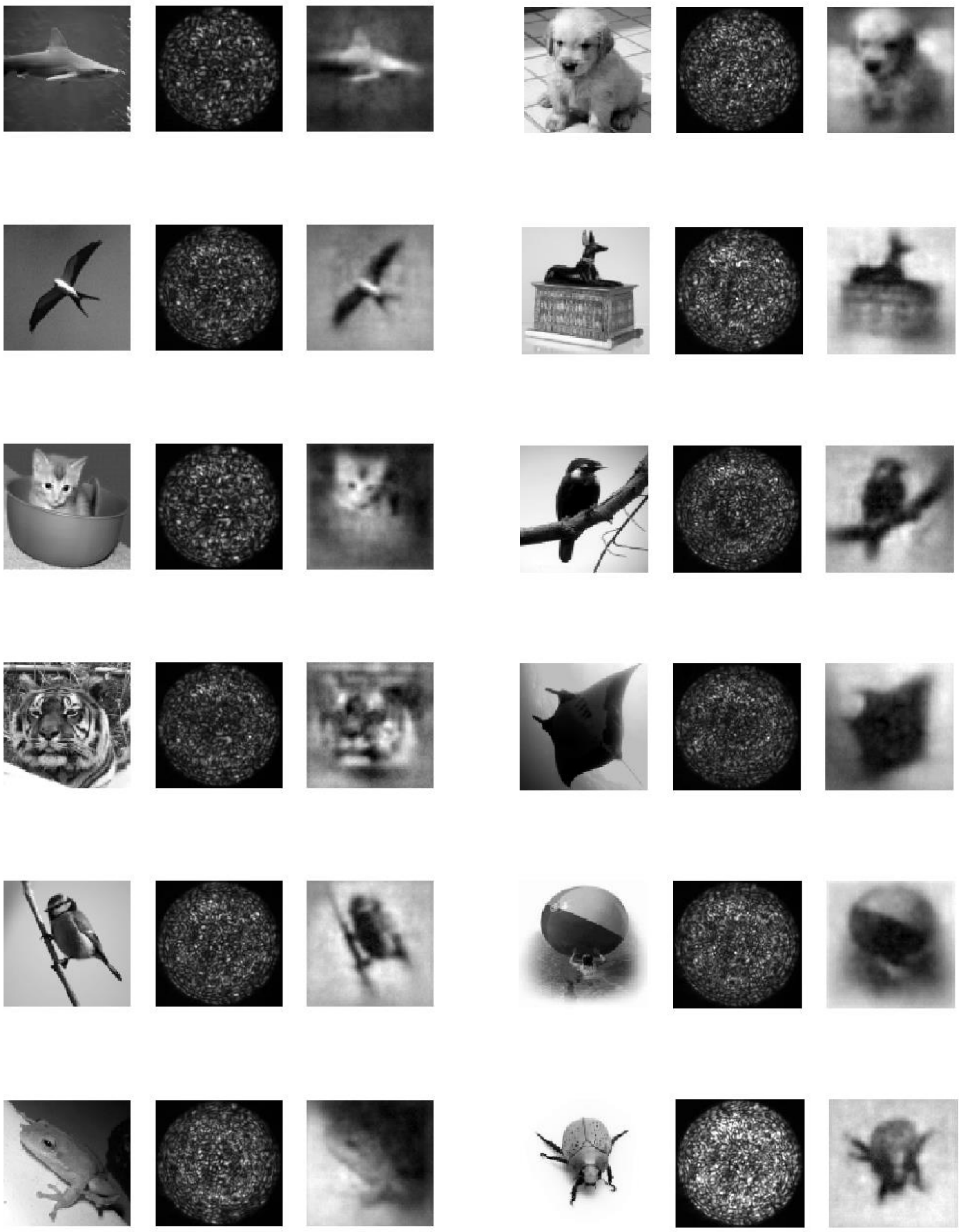

Supplementary Figure 3. A sample collection of images from the ImageNet database, as projected into the fibre together with their relative output speckle patterns and retrieved images. These testing images were not present in the training dataset.

SUPPLEMENTARY REFERENCES

[1] Papadopoulos, I. N., Farahi, S., Moser, C. & Psaltis, D. Focusing and scanning light through a multimode optical fiber using digital phase conjugation. *Optics express* **20**, 10583–10590 (2012).
[2] Wang, Z., *et al.* Image quality assessment: from error visibility to structural similarity. *IEEE transactions on image processing* **13**, 600–612 (2004).
[3] Deng, J. *et al.* ImageNet: A Large-Scale Hierarchical Image Database. In *CVPR09* (2009).
[4] Caramazza, P., Moran, O., Murray-Smith, R. & Faccio, D. *Data can be downloaded from the University of Glasgow repository:* URL `DOI:http://dx.doi.org/10.5525/gla.researchdata.751`.
[5] Chollet, F. *et al.* Keras. `https://keras.io` (2015).
[6] Abadi, M. *et al.* TensorFlow: Large-scale machine learning on heterogeneous systems (2015). URL `https://www.tensorflow.org/`. Software available from tensorflow.org.
