# Supplementary material for: Transmission of natural scene images through a multimode fibre
Source: Nat Commun. 2019 May 2;10:2029. doi: 10.1038/s41467-019-10057-8 (PMC6497636; doi:10.1038/s41467-019-10057-8)
Supplement: Supplementary file 3 — Description of Additional Supplementary Files [file 41467_2019_10057_MOESM3_ESM.docx]

**Description of Supplementary Files**

**File Name:** Supplementary Video 1

**Description:** Full Muybridge videos reconstruction relative to Figure 2.
